# Antioxidant, α-Glucosidase, and Nitric Oxide Inhibitory Activities of Six Algerian Traditional Medicinal Plant Extracts and ^1^H-NMR-Based Metabolomics Study of the Active Extract

**DOI:** 10.3390/molecules25051247

**Published:** 2020-03-10

**Authors:** Khaoula Hellal, M. Maulidiani, Intan Safinar Ismail, Chin Ping Tan, Faridah Abas

**Affiliations:** 1 Department of Food Science, Faculty of Food Science and Technology, Universiti Putra Malaysia, Serdang 43400, Selangor, Malaysia; khaoulahellal@gmail.com; 2 School of Fundamental Science, Universiti Malaysia Terengganu, Kuala Nerus 21030, Terengganu, Malaysia; maulidiani@umt.edu.my; 3 Laboratory of Natural Products, Institute of Bioscience, Universiti Putra Malaysia, Serdang 43400, Selangor, Malaysia; safinar@upm.edu.my; 4 Department of Chemistry, Faculty of Science, Universiti Putra Malaysia, Serdang 43400, Selangor, Malaysia; 5 Department of Food Technology, Faculty of Food Science and Technology, Universiti Putra Malaysia, Serdang 43400, Selangor, Malaysia; tancp@upm.edu.my

**Keywords:** *Lupinus albus*, medicinal plants, diabetes, α-glucosidase, metabolomics

## Abstract

Claims of effective therapy against diabetes using plants including *Peganum harmala* L., *Zygophyllum album, Anacyclus valentinus* L., *Ammodaucus leucotrichus, Lupinus albus*, and *Marrubium vulgare* in Algerian empirical medicine prompted our interest in evaluating their antidiabetic activity by screening their free radical scavenging (DPPH), α-glucosidase, and nitric oxide (NO) inhibitory activities as well as the total phenolic content (TPC). Extracts of the selected plants were prepared using different ratios of ethanol (0, 50, 80, and 100%). In this study, 100%, and 80% ethanol extracts of *L. albus* were found to be the most potent, in inhibiting α-glucosidase activity with IC_50_ values of 6.45 and 8.66 μg/mL, respectively. The 100% ethanol extract of *A. leucotrichus* exhibited the highest free radical scavenging activity with an IC_50_ value of 26.26 μg/mL. Moreover, the highest TPC of 612.84 μg GAE/mg extract was observed in *M. vulgare*, extracted with 80% ethanol. Metabolite profiling of the active extract was conducted using ^1^H-NMR metabolomics. Partial least square analysis (PLS) was used to assess the relationship between the α-glucosidase inhibitory activity of *L. albus* and the metabolites identified in the extract. Based on the PLS model, isoflavonoids (lupinoisoflavone G, lupisoflavone, lupinoisolone C), amino acids (asparagine and thiamine), and several fatty acids (stearic acid and oleic acid) were identified as metabolites that contributed to the inhibition of α-glucosidase activity. The results of this study have clearly strengthened the traditional claim of the antihyperglycemic effects of *L. albus.*

## 1. Introduction

Diabetes mellitus (DM) is the most widely recognized endocrine disorder in almost all countries. According to the World Health Organization (WHO) (2016), an estimated 1.6 million deaths were directly caused by diabetes. Due to the adverse effects of modern treatment such as hypoglycemic coma, renal complications, stomach upset, and weight gain, the use of medicinal plants as antidiabetic agents has been gaining increasing attention due to their effectiveness. The latter is strongly supported by several empirical therapies and clinical experiences accumulated over hundreds of years [1]. In addition, due to other historical, cultural, and economic reasons, people are diverging toward herb-based drugs and natural sources to help in the treatment of several diseases including diabetes [2]. Arid areas are a good source of medicinal herbs; therefore, Algeria is fortunate to have a variety of flora that are rich in therapeutic and aromatic plants. Moreover, several of these plants have not yet been evaluated scientifically for their abilities to treat or prevent diabetes [1,2]. In this study, the plants *Peganum harmala* L., *Zygophyllum album*, *Anacyclus valentinus* L., *Ammodaucus leucotrichus*, *Lupinus albus*, and *Marrubium vulgare* were selected on the basis of their ethnomedical records as antidiabetic agents and also due to the fact that they might contain several bioactive compounds against DM [1].

*P. harmala* L. (family Nitrariaceae) is also known as Harmal or Suryin Rue [3]. Current biological studies have reported that *P. harmala* possesses analgesic and anti-inflammatory properties [4], wound healing and antioxidant [5], and hypoglycemic [6] activities. The active constituents of *P. harmala* are alkaloids that are found especially in the seeds and roots. These include β-carbolines such as harmine and harmaline (identical with harmidine), harmalol, harman, and the quinazoline derivatives; and vasicine and vasicinone [6]. In folk medicine, *Z. album* (family Zygophyllaceae) has been used as a diuretic, local anesthetic, antihistaminic, and antidiabetic agent [7]. Phytochemical screening results of *Z. album* have demonstrated the presence of alkaloids, flavonoids, and saponins as the major compounds. Furthermore, it has been reported that *Z. album* contains glycosides, coumarins, sterols and/or triterpenes, tannins, and cardiac glycosides [8]. *A. valentinus* L. (family Asteraceae), also known as “Gartoufa”, is a herb used by the Saharan population of Algeria for treating several diseases including diabetes. Phytochemical analysis of the aqueous extract of this species demonstrated the presence of a variety of compounds such as tannins, saponins, flavonoids, cardiac glycosides, coumarins, alkaloids, mucilage, and amino acids [9]. *A. valentinus* possesses significant antidiabetic activity and the crude aqueous extract also contains certain mineral elements such as K and Ca, which have been claimed to participate in insulin secretion [10]. *A. leucotrichus* (family Apiaceae), commonly known as “Hairy Cumin’’, is widely used in North African countries as a condiment or spice and in traditional medicine as a cure for cold, fever, and digestive disorders, particularly in children [11]. A few studies have analyzed the phytochemistry of *A. leuchotrichus*, but with the exception of a guaianolide and amolactone, there is not much information available concerning the chemical constituents of *A. leuchotrichus* [11].

*M. vulgare* (family Lamiaceae) is known by the name Marriouth in Algeria; it has been frequently used in folk medicine to treat a variety of maladies. The plant has been reported to possess hypoglycemic [12], anti-inflammatory, antispasmodic, anti-nociceptive, and several other reported biological activities [13]. Phytochemicals present in the plant include caryophyllene oxide and trans-caryophyllene, caffeoyl-l-malic acid, acteoside, phenylethanoid glycoside, marruboside [13], and vulgarol, β-sitosterol, lupeol, and marrubiin [14]. The seeds of *L. albus* (family Fabaceae) are also known to possess diuretic, emmenagogue, hypoglycemic, and vermifuge activities [15], and according to Schwartz (1906), these seeds contain a crystalline substance known as magolan, which is a useful remedy for treating diabetes mellitus [15]. *L. albus* is also a rich source of proteins (33–47%), oils (6–13%), and contain a high concentration of polyunsaturated fatty acids and fibers [16]. These primary components are extremely valuable and make *L. albus* an important crop for human nutrition.

Nevertheless, no sufficient data are currently available regarding the effect of binary solvents on the biological activity of the six Algerian medicinal plants that are traditionally used for the treatment of hyperglycemia (*P. harmala* L., *Z. album*, *A. valentinus* L., *A. leucotrichus*, *L. albus*, and *M. vulgare).* Therefore, this comparative study was conducted to fill in the current research gap existing for these plants. The total phenolic content (TPC), 2,2-diphenyl-2-picrylhydrazyl (DPPH), α-glucosidase, and nitric oxide (NO) inhibitory activities were evaluated in this study. In addition, the nuclear magnetic resonance (NMR) metabolomics approach was applied to identify the potential bioactive compounds present in the most active extract. To the best of our knowledge, this study provides the first detailed metabolite composition and correlation with the biological activities of *L. albus* by using a NMR-based metabolomics approach.

## 2. Results and Discussion

### 2.1. Effect of Ethanol Ratios on the Total Phenolic Content of Selected Plant Extracts

**Figure 1:**
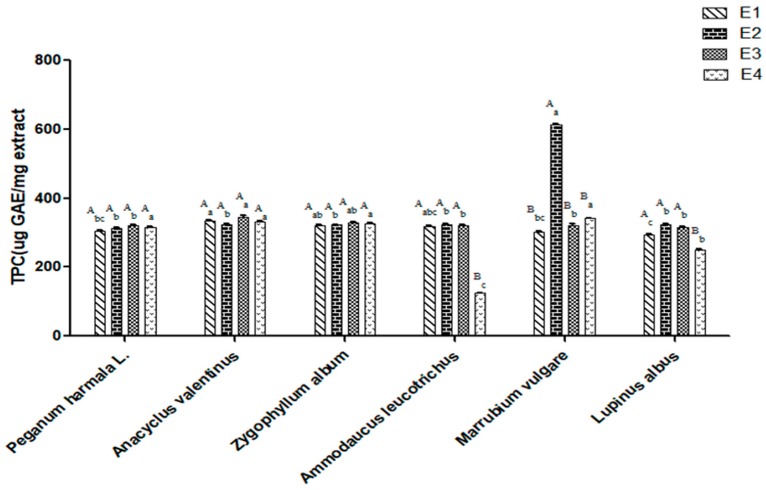
Total phenolic content of the six Algerian plants extracted by four ethanol ratios. (**E1**) 100% ethanol extracts; (**E2**) 80% ethanol extracts; (**E3**) 50% ethanol extracts; (**D**) aqueous extracts. Values are the means ± standard deviation based on six replicates. The subscripts represent the comparison between different ethanol ratios in the same species. The superscripts represent the comparison between the same ethanol ratios in the different species. Means with different subscripts and superscripts letters are significantly different (*p* < 0.05).

Phenolic compounds have a significant capacity for adsorbing and neutralizing free radicals, quenching singlet oxygen, or decomposing peroxides because of the presence of hydroxyl substituents and their aromatic structure, which allows them to scavenge free radicals [17]. Phenolic compounds are also well known due to a variety of biological properties such as antiallergenic, antiatherogenic, anti-inflammatory, antimicrobial, antithrombotic, cardioprotective, and vasodilatory effects [18]. The total phenolic content of the six selected plants are presented in Figure 1.

There was no statistically significant difference between the four solvent ratios in every species (*p* > 0.05), whereas a significant difference (*p* < 0.05) was observed between the same solvent ratio of the six species as shown by the results in Table 1. The findings clearly indicated that the values ranged between 124.98 and 346.05 μg GAE/mg extract, with a noticeable close value in almost all extracts. This result may be related to the solvent system used in this study, since the phenolic content in some of the extracts was dependent on the polarity of the solvent used [19]. Ethanol and aqueous ethanol were found to be more efficient and non-toxic in the extraction of lower molecular weight polyphenols. Overall, the polarity of the solvent system used in this study (ethanol and water) was still considered to have a high polarity to extract the phenolic compounds that have a high solubility in polar solvents; these results are supported by a previous study conducted by Stankovic where methanol and water extracts yielded the same phenolic content [20]. Unlike *P. harmala* L., *Z. album*, and *A. valentinus* L., the effect of the solvent on the phenolic content was noticeable in *A. leucotrichus*, *L. albus*, and *M. vulgare*. The high ethanol ratio mixtures could be associated with the solubility factor of the phenolic compounds rather than the water mixtures, as observed by the highest value shown by *M. vulgare* extracted using 80% ethanol (612.84 μg GAE/mg extract). This finding is consistent with those reported by Amri et al. [21], where an 80% ethanolic extract of *M. vulgare* was used for the same assay. On the other hand, *A. leucotrichus* showed the least amount of phenolic compounds among the samples tested, with a value of 124.98 μg GAE/mg extract, which may indicate the type of phenolic compounds represented in *A. leucotrichus* that are insoluble in water [22]. Generally, due to the differences that can appear at the level of the extraction methodology, the polarity of the extracting solvent, and the method, we may notice differences in the findings obtained in this study compared with the previous results.

### 2.2. Effect of Ethanol Ratios on the Free Radical Scavenging Activity

Antioxidants play a vital role as health protecting factors. Scientific evidence claims that antioxidants can reduce the risk of developing chronic diseases such as diabetes [23]. There are several forms of antioxidants including plant-derived polyphenols; the antioxidant neutralizes free radicals either by providing the additional electron required to make the pair, or by breaking down the free radical molecule to render it harmless [24]. 2,2-Diphenyl-1-Picrylhydrazyl (DPPH) free radical scavenging activity was evaluated to determine the antioxidant activity of the selected plant species and the results were expressed as the IC_50_ value. IC_50_ is defined as the concentration of the antioxidant required to scavenge 50% of the DPPH radical.

According to the findings shown in Table 1, a significant difference (*p* < 0.05) was noticed between the DPPH free radical scavenging activities of the four solvent ratio extracts in the same species and the six species extracted using the same solvent ratio. The highest free radical scavenging inhibition activity was achieved with the 100% ethanolic extract of *A. leucotrichus* (81.60% ± 0.56) with an IC_50_ value of 26.26 μg/mL. This value was almost similar to that reported by Sebaa et al. [25], who used a 100% methanolic extract of the fruits of A. *leucotrichus* that provided an inhibitory activity of 93.31% ± 4.87, in contrast to the results reported by Dahmane et al. [26], who reported that *A. leucotrichus* possessed weak antioxidant activity. It should be emphasized that since all previous studies have only investigated the activity of the essential oil and due to the lack of studies on the free radical scavenging activity of the crude extract of *A. leucotrichus*, an appropriate comparison could not be made between the results.

In addition to *A. leucotrichus*, the 100% and 80% ethanolic extracts of *M. vulgare* exhibited high activities of 75.84% ± 2.95 and 78.57% 1.88, respectively, with IC_50_ values of 24.08 and 19.67 μg/mL respectively. These values were found to be higher than the results reported by Pukalskas et al. [27] where the 80% methanolic extract of *M. vulgare* showed a free radical scavenging activity of 58.20% ± 0.7. On the other hand, *A. valentinus* was screened for the first time, which resulted in a moderate activity of 65.57% ± 1.32 with an IC_50_ value of 25.17 μg/mL by the 80% ethanolic extract. This result was higher than that observed with *Anacyclus clavatus*, a species of the same genus that exhibited its activity with an IC_50_ value of 54.77 μg/mL [28]. From the other hand, the result was lower than that observed with the methanolic extract of *Anacyclus pyrethrum*, another species of the same genus, which exhibited high activity with an IC_50_ value of 5.60 μg/mL [29]. Its noteworthy to highlight that the IC_50_ of some extracts was not determined due to the samples’ low activities, which were less than 50% inhibition at 5000 μg/mL.

Various studies have outlined the strong relationship between the phenolic content and the antioxidant activity [30]; however, other researchers have denied this relationship [31]. Overall, the correlation coefficient between the total phenolics and the antioxidative activities was statistically significant. Our results did not reveal any relationship between the antioxidant activity and the total phenolic content; for example, *M. vulgare* had the highest total phenolic content, whereas its antioxidant activity was lower than that reported of *A. leucotrichus*; which exhibited a very low value of total phenolic content compared to that with *M. vulgare*. This can be due to the type of phenolics and the amount of the individual phenolic compounds present in the sample, or to the high flavonoid content of the sample. Ismail et al. [31] reported that due to the chemical structure of flavonoids with more hydroxyl groups and the ortho-substitution with electron-donating alkyl or methoxy groups of flavonoids, the stability of the free radical increased, and therefore its antioxidant potential. In general, the results found in this study clearly indicate that every sample had a different antioxidant activity contributed by different antioxidant compounds; these compounds react differently depending on the extraction conditions and the polarity of the extracting solvent. In addition, these results suggested that high solvent ratios (80% and 100%) were the most efficient in the extraction process, which might be due to their ability to breakdown the plant cell wall and release more antioxidant compounds [32].

### 2.3. Effect of Ethanol Ratios on the α-Glucosidase Inhibitory Activity

Based on the results shown in Table 1, a significant difference (*p* < 0.05) was observed between the α-glucosidase inhibitory activities of all of the samples. The 100% ethanolic extract of *L. albus* showed the maximum percentage inhibition of 96.78% ± 1.75 with an IC_50_ value of 6.45 μg/mL, which was higher than the inhibition of the 80% ethanolic extract of the same species of 64.57% ± 1.96 with an IC_50_ of 8.66 μg/mL; the α-glucosidase inhibitory activity of *L. albus* in this study was directly proportional to the increase in ethanol ratio. Piślewska et al. [33] highlighted that isoflavones and their glucosides were concentrated in the cell walls of *L. albus*; the capability of absolute ethanol in liquefying the chemical composites may be related to the degradation of the plant cell wall, thereby allowing the extraction of more endocellular compounds [34]. This may explain the high α-glucosidase inhibitory activity of high ethanol ratio extracts of *L. albus*. Moreover, the 80% ethanol extract of *M. vulgare* exhibited an acceptable activity of 68.06% ± 2.15 with an IC_50_ value of 12.6 μg/mL. Despite the strong hypoglycemic potential that was highlighted by the wide use of these plants in Algerian folk medicine and the fact that previous studies have reported these species were a natural and infinite source of the phenolic compounds as well as the flavonoids. In addition, no significant activity was exhibited by any of the remaining samples including *P. harmala*, *Z. album*, *A. valentinus*, and *A. leucotrichus*. This finding was consistent with the results reported by [35]. As mentioned earlier, the IC_50_ of some extracts was not determined due to their low inhibition activity at the highest concentration (5000 μg/mL).

### 2.4. Effect of Ethanol Ratios on the Nitric Oxide Inhibitory Activity

Nitric oxide performs several functions including the modulation of inflammatory responses. This assay determines the nitric oxide concentrations based on the enzymatic conversion of nitrate into nitrite by nitrate reductase. This assay is useful in monitoring individual condition and provides a measure of the innate immune response. All of the plant species in this study did not exhibit any significant nitric oxide inhibitory activity, as indicated by their low values, which were measured via the Griess assay. The highest nitric oxide inhibitory activity values were observed with 100% ethanol extracts of *A. leucotrichus* with 50.53% and *M. vulgare* with 49.87%, which have been reported for their weak inhibitory activity for NO by Hammami et al. [36]. The other species selected in this study were screened for the first time for their nitric oxide inhibitory activity, and there are no reports in the literature in this context.

### 2.5. Identification of the Metabolites from L. Albus Extract

**Figure 2:**
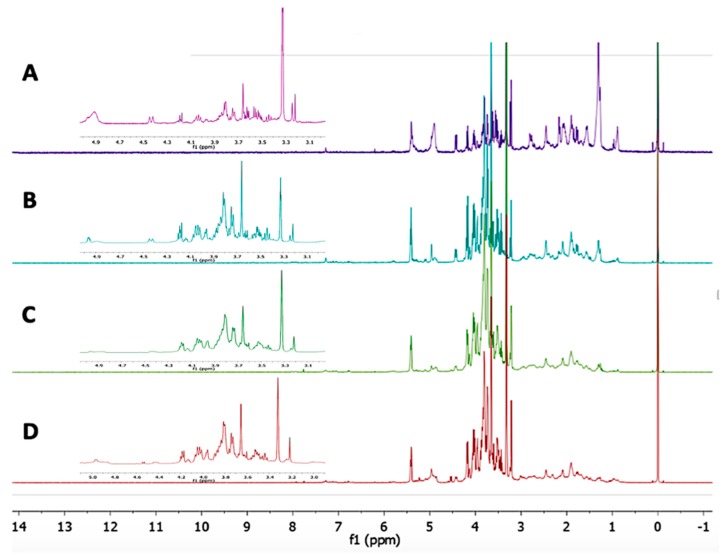
Representative ^1^H-NMR spectra of the four different ethanol ratios of *L. albus* extracts. (**A**) Spectra of 100% ethanol extract of *L. albus*; (**B**) Spectra of 80% ethanol extract of *L. albus*; (**C**) Spectra of 50% ethanol extract of *L. albus*; (**D**) Spectra of aqueous extract of *L. albus.*

According to the results and on the basis of the important α-glucosidase inhibitory activity value, *L. albus* was the best candidate among the selected plants for further study using ^1^H-NMR metabolomics. The identification of the metabolites in the *L. albus* extract was primarily based on the 2D-J-resolved spectra, the Chenomx database, and comparison with the NMR signals of standard references. The ^1^H-NMR spectrum of *L. albus* contains a multitude of overlapping signals, with multiple signals from each detected metabolite. Figure 2 illustrates the spectra of the four ethanolic extracts of *L. albus* (0, 50, 80 and 100%), which clearly shows that the intensity of peaks in the aliphatic region (δ 0.5–3.5) of the 100% ethanol extract was higher than those obtained with 80%, 50%, and 0% ethanolic extracts. In contrast to the aliphatic region, the intensity of peaks in the carbohydrate region (δ 3.5–5.5) was the lowest among the four spectra.

As presented in Table 2, a total of 34 metabolites were identified in the ethanolic extract of *L. albus*. Flavonoids were also identified such as wighteon at (δ 6.49 (s), δ 5.28 (br t), δ 3.37 (br d), δ 1.78 (s), 1.65 (s)); kaempferol at (δ 8.02 (d, *J* = 8.0 Hz), δ 6.92 (d, *J* = 8.0 Hz), δ 6.35 (d, *J* = 2.0 Hz), δ 6.20 (d, *J* = 2.0 Hz)); and (epi)catechin at (δ 7.05 (br s), δ 6.96 (m), δ 6.12 (d, *J* = 1.5 Hz), δ 6.08 (d, *J* = 1.5 Hz), δ 5.01 (br s), δ 2.94 (m), δ 2.78 (m)). The characteristic signals of some isoflavonoids that were practically found in *L. albus* were successfully detected at the level of the aromatic region including, lupisoflavone (δ 7.27 (d, *J* = 2.0 Hz), δ 6.88 (d, *J* = 8.2 Hz), and δ 3.88 (m)); lupinoisolone A (δ 13.40 (s), δ 8.18 (s), δ 6.33 (s), δ 5.33 (brt, *J* = 7.0 Hz), δ 3.89 (m), δ 1.70 (brs), δ 1.25 (brs)); lupinisolone C (δ 13.41 (s), δ 7.94 (s), δ 7.03 (d, *J* = 8.2 Hz), δ 6.44 brs, δ 6.41 (d, *J* = 8.0 Hz), δ 3.75 (brt, *J* = 7.0 Hz), δ 1.62 (s), δ 1.28 (s)); lupinisol A (δ 13.58 (s), δ 8.12 (s), δ 6.45 (s), δ 7.33 (s), δ 7.29 (dd, *J* = 8.3, 2.0 Hz), δ 6.89 (d, *J* = 8.3 Hz), δ 5.35 (br t, *J* = 7.0 Hz), δ 4.72 (brs), δ 4.4 (m), δ 3.35 (br d, *J* = 7.0 Hz)); lupinisol B (δ 13.00 (s), δ 8.18 (s), δ 6.95 (d, *J* = 8.0 Hz), δ 6.52 (s), δ 5.32 (br t, *J* = 7.0 Hz), δ 4.94 (br s), δ 4.77 (br s), δ 4.48 (dd, *J* = 7.0, 4.0 Hz), δ 3.48 (br d, *J* = 5.0 Hz)); lupinisol C (δ 13.01 (s), δ 8.14 (s), δ 7.05 (d, *J* = 7.0 Hz), δ 6.56 (s), δ 5.28 (br t, *J* = 7.3 Hz), δ 5.04 (br s), δ 4.85 (br s), δ 4.37 (br d, *J* = 5.0 Hz), δ 3.38 (br d, *J* = 5.0 Hz)); chandalone (δ 13.46 (s), δ 8.14 (s), δ 7.35 (d, *J* = 2.0 Hz), δ 7.29 (dd, *J* = 2.0, 7.0 Hz), δ 6.88 (d, *J* = 2.0 Hz), δ 6.69 (d, *J* = 9.0 Hz)); isoderrone (δ 13.00 (s), δ 8.25 (s), δ 7.29 (d, *J* = 2.0 Hz), δ 6.48 (d, *J* = 2.0 Hz), δ 6.44 (d, *J* = 1.5 Hz), δ 6.31 (d, *J* = 1.5 Hz), δ 5.77 (d, *J* = 5.0 Hz), δ 1.41 (s)); lupinalbin F (δ 13.58 (s), δ 8.12 (s), δ 7.33 (s), δ 7.29 (dd, *J* = 8.3, 2.0 Hz, δ 4.95 (br s), δ 4.40 (m), δ 3.35 (br d, *J* = 7.0 Hz)); and lupinoisoflavone G (δ 8.14 (s), δ 7.35 (d, *J* = 2.0 Hz), δ 7.29 (dd, *J* = 8.0, 2.0 Hz), δ 6.37 (s), δ 5.33 (br t, *J* = 7.1 Hz), δ 3.18 (br d, *J* = 8.0 Hz), δ 1.73 (br s), δ 1.29 (s), δ 1.25 (s)) [37,38].

The primary and secondary metabolites were assigned and confirmed by a comparison of the data and literature review including amino acids such as asparagine (δ 4.02 (dd, *J* = 7.5, 4 Hz), δ 2.96 (m), δ 2.88 (m)); thiamine (δ 5.47 (s), δ 3.87 (t, *J* = 5.8 Hz), δ 3.20 (t, *J* = 5.5 Hz), δ 2.55 (d, *J* = 15.5 Hz), and proline (δ 2.33 (m), δ 4.11 (m), δ 3.35 (m), δ 3.30 (m)) [39,40]. In addition, several fatty acids were detected, including stearic acid at (δ 2.42), oleic acid at (δ 2.02), palmitic acid at (δ 3.87), and linoleic acid at (δ 2.3) and triterpenoids; oleanolic acid at (δ 1.3), betulinic acid at (δ 4.94), and lupeol at (δ 1.64), which are consistent with the data reported by Mohamed and Rayas-Duarte [41].

### 2.6. Multivariate Data Analyses

**Figure 3:**
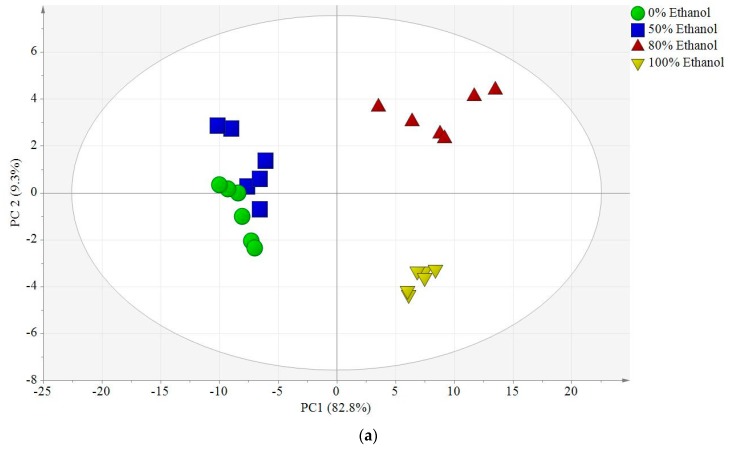

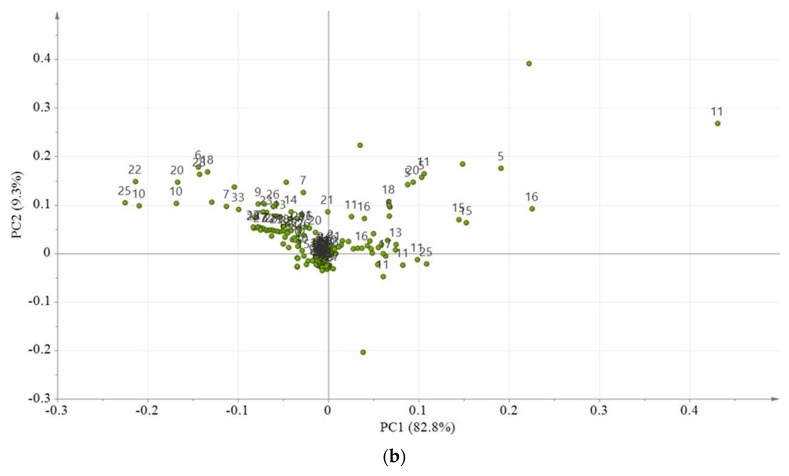
(**a**) Principal component analysis score plot. (**b**) Loading scatter plot of the four extracts of *L. albus* with different solvent ratios (100% ethanol, 80% ethanol, 50% ethanol, and water).

Principal component analysis (PCA) and partial least square analysis (PLS) plots were obtained from the different ethanolic extracts of *L. albus*; these samples were used for this analysis based on their high α-glucosidase inhibitory activity. As shown in Figure 3a, the PCA scores revealed the existence of three well-separated clusters without any significant outliers. PC1 clearly exhibited the most sample variation of 82.8%, separating the 100% and 80% ethanolic extracts from 50% ethanol and the aqueous extracts, whereas PC2 displayed a variance of 9.3%. This separation was due to the variation in metabolites between the four ethanolic extracts as suggested by the loading plot in Figure 3b. The PCA and the loading plot were complementary to each other, and the metabolites comprising wighteon, lupinisol A, lupinoisoflavone G, lupisoflavone, lupinoisolone A, hydroxyiso-lupalbigenin, adenosine, asparagine, kaempferol, linoleic acid, methoxyisoflavone, and alpha-tocopherol were predominant in the 100% and 80% ethanol extracts of *L. albus* when compared to those in the 50% and 0% ethanol extracts. However, compounds such as hydroxyiso-lupalbigenin, lupinisol B, palmitic acid, lupeol, and epicatechin had a higher content in the 50% and 0% ethanolic extracts.

**Figure 4:**
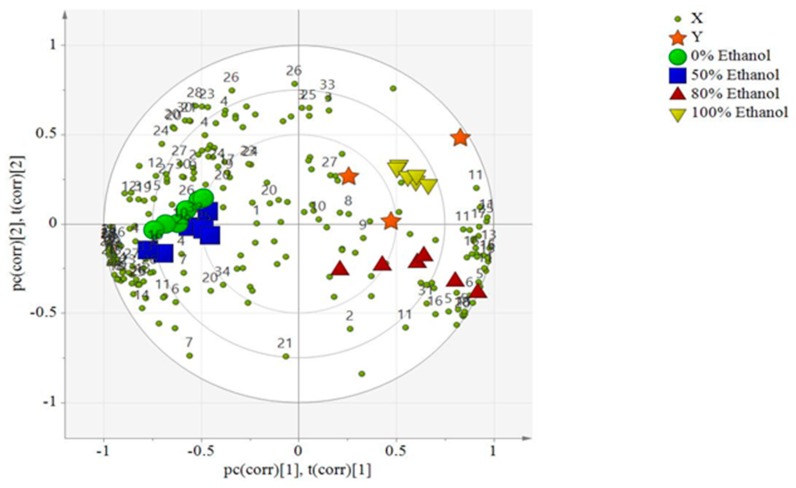
The Partial least squares (PLS) biplot showed the correlation of metabolite variations with the selected test bioactivities in the *L. albus* extracts. The numbering of signals is according to the metabolites listed in Table 2.

Partial least squares (PLS) biplot analysis was conducted to correlate the α-glucosidase, DPPH, and NO inhibitory activities with the phytochemical contents of the four ethanolic extracts of *L. albus*. The PLS biplot for the 1H-NMR data of the *L. albus* ethanolic extracts (Figure 4) revealed an obvious separation into four different clusters. PC1 separated the 100% and 80% ethanolic extracts from the 50% ethanolic and water extracts, while the 100% ethanolic and water extracts were separated from the 80% and 50% ethanolic extracts by PC2. The findings demonstrated that the 100% ethanolic extract of *L. albus* was strongly correlated with α-glucosidase inhibition activity, followed by the 80% extract, which was also confirmed by the bioassay results.

However, the clusters of the 50% ethanolic and water extracts, which did not exhibit any significant activity, were grouped separately in the opposite and negative side, presenting a negative correlation with the bioactivities. This dissimilarity may be attributed to the effect of the ethanol ratio in the extraction of the chemical compounds that contributed to this differentiation between the extracts of *L. albus*.

The Fabaceae family has been shown to possess a wide range of bioactive metabolites including saponins and phenolic compounds [42]. Within this large family, isoflavones belong to a subclass that are produced almost exclusively by the species of Fabaceae, and they also strikingly possess several bioactivities just like flavonoids [43]. In this context, considerable studies have shown that a combination of flavonoids (kaempferol and epicatechin) including isoflavonoids (lupinoisolone A, lupinoisolone C, lupinisol A, and lupinoisoflavone G) and fatty acids were strongly contributing to the antioxidant activity of *L. albus* [44]. Furthermore, it has clearly been shown that primary metabolites such as alpha-tocopherol and amino acids (asparagine, thiamine, proline) that are present in high amounts in *L. albus* seeds act as antioxidants in oils and fats [45]. In addition, some studies have reported that some flavonoids are effective α-glucosidase inhibitors such as kaempferol [46]. All these findings may explain the strong α-glucosidase inhibitory activity of *L. albus* (100%, and 80% EtOH extracts).

The variable importance to projections (VIP) was acquired to measure the contribution that a variable makes to the model [47]. A region of VIP > 1 indicates the compounds that can be distinguished as important for optimal PLS model performance. In this case, compounds including alpha-tocopherol, proline, caprate, lupinioisolone C, lupinalbin F, lupinoisoflavone G, betulinic acid, oleanolic acid, luteone, lupinoisolone A, and lupinisol B can be selected for optimal performance. The PLS model exhibited a perfect goodness of fit where (R2Y (cum) > 0.9) and predictive quality (Q2(cum) > 0.9). Results showed that the *Y*-axis intercepts of R2 and Q2 for the three assays were in the range of 0.28–0.56 and −0.4 to −0.42, respectively. A permutation test with 200 random permutations was performed to confirm the validity of the PLS biplot model with the goodness of fit of several models as well as the predictive ability of the model (R2/Q2).

### 2.7. Relative Quantification of Metabolites

**Figure 5:**
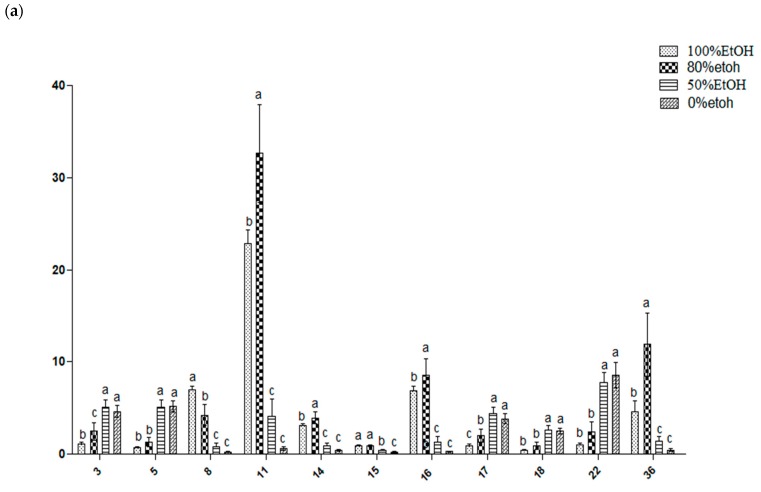

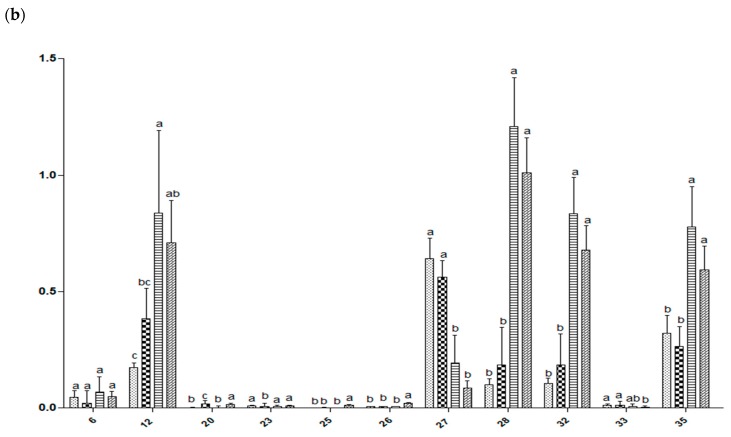
Relative quantification of the identified metabolites in *L. albus* extracts with different ethanolic solvent ratios. (**a**) 3, hydroxybutyrate (δ 3.58); 5, proline (δ 3.3); 8, caprate (δ 0.86); 11, alpha-tocopherol (δ 1.26); 14, oleic acid (δ 5.35m); 15, linoleic acid (δ 2.3); 16, oleanolic acid (δ 1.3); 17, betulinic acid (δ 3.54); 18, lupeol (δ 3.22); 22, luteone (δ 3.44); 36, unknown (δ 1.24). (**b**) 6, thiamine (δ 5.46); 12, stearic acid (δ 2.42); 20, hydroxyiso-lupalbigenin (δ 6.66); 23, lupisoflavone (δ 6.86); 25, Lupinoisolone A (δ 6.33); 26, Lupinoisolone C (δ 13.41); 27, lipinisol A (δ 5.38); 28, lupinisol B (δ 4.46); 32, lupinalbin F (δ 4.74); 33, lupinoisoflavone G (δ 6.38); 35, unknown (δ 5.22). Values are expressed as the mean ± standard deviation (n = 6). Means with different letters are significantly different (*p* ≤ 0.001).

Based on the PLS model, the relative quantification of the metabolites that possessed a high value (>1) in the projection (VIP) was performed to further validate whether these metabolites affected the discrimination among samples. Figure 5 shows the relative quantification of the metabolites in the different ethanolic extract of *L. albus*. Overall, a high significant difference (P ≤ 0.001) was observed in the metabolites of the 50% ethanolic and aqueous extracts, which were found to contain a higher amount of thiamine (δ 5.46), proline (δ 3.3), lupeol (δ 3.22), stearic acid (δ 2.42), betulinic acid (δ 3.54), luteone (δ 3.44), and lupinoisoflavone G (δ 6.38). These extracts also had a high polar solvent ratio (80%, and 100%), which was characterized by a significantly higher concentration of alpha-tocopherol that was detected at (δ 1.26), unknown 35 (δ 1.24), oleanolic acid (δ 1.3), linoleic acid (δ 2.3), caprate (δ 0.86), and lipinisol A (δ 5.38). These findings were consistent with the PLS results (Figure 3), which suggested that these metabolites contributed to the clustering of the metabolites and influenced the bioactivities of the extracts.

## 3. Materials and Methods

### 3.1. Chemicals and Reagents

Folin-Ciocalteu reagent, sodium carbonate (Na_2_CO_3_), gallic acid (purity of 97%), quercetin (purity of >95%), curcumin (purity of >98%), phosphate buffer, α-glucosidase enzyme, glycine, 2,2-diphenyl-2-picrylhydrazyl (DPPH), and p-nitrophenyl- α-D-glucopyranoside (PNPG) were supplied by Sigma-Aldrich (St. Louis, MO, USA). Absolute ethanol was purchased from Merck Millipore International (Darmstadt, Germany). The reagents used for cell culture including media Dulbecco’s Modified Eagle’s Medium (DMEM) containing 4-(2-hydroxyethyl)-1-piperazineethanesulfonic acid (HEPES) and L-glutamine (with and without phenol red), penicillin-streptomycin antibiotic solution, fetal bovine serum (FBS), 3-(4,5-dimethylthiazol-2-yl)-2,5-diphenylte-trazolium bromide (MTT), and TrypLE Express enzyme were obtained from Gibco/BRL Life Technologies Inc. (Eggenstein, Germany). Recombinant murine IFN-γ, lipopolysaccharide (LPS), and phosphate-buffered saline (PBS) were supplied by Sigma-Aldrich Co. (St. Louis, MO, USA). Dimethyl sulfoxide-d6 (DMSO) and trimethylsilyl propionic acid-d4 sodium salt (TSP) were purchased from Merck Millipore International (Darmstadt, Germany). Water was purified by a MiliQ system (Millipore, Bedford, MA, USA).

### 3.2. Plant Materials

All samples were harvested during the summer of 2015 from various areas of Algeria. The aerial parts of *P. harmala* L. and *M*. *vulgare* were collected from Sétif (Eastern Algeria). However, the aerial parts of *Z. album*, *A. valentinus* L., *A. leucotrichus*, and the seeds of *L. albus* were collected from the Saharian region (Southwestern Algeria). The plants used in this study were identified by an in-house botanist (Dr. Idder Boubaker) at the Department of Microbiology and Plant Biology, University of Abdelhamid Ibn Badis, Mostaganem, Algeria. The vouchers specimens *P. harmala* (LMPB-0015-2019), *M*. *vulgare* (LMPB-0014-2019), *Z. album* (LMPB-0013-2019), *A. valentinus* (LMPB-0018-2019), *A. leucotrichus* (LMPB-0016-2019), and *L. albus* (LMPB-0017-2019) were deposited in the herbarium at the University. All samples were cleaned from any impurities with clean tissue before air-dried at ambient temperature until a constant weight was achieved. The dried samples were ground using a laboratory blender (Waring Commercial, Torrington, CT, USA).

### 3.3. Plant Extraction

To produce the crude extract, 4 g of powdered samples were soaked in 100 mL of different ratios of ethanol (0, 50, 80 and 100%). The mixtures were subjected to sonication for 1 h in an ultrasonic bath sonicator (Branson, 141 8510E-MTH model, Danbury, NC, USA). The ultrasonic was operated at a power voltage of 220 V and frequency of 53 kHz. Each mixture was filtered thrice using filter paper; this extraction procedure was repeated six times for each solvent ratio, with a total of 24 extracts for each species. The filtered extracts were collected and concentrated using a rotary evaporator under vacuum at 40 °C to yield the crude extract, which was subjected to freeze drying to ensure that no water remained. The extracts obtained were stored in amber bottle at 4 °C until further analysis.

### 3.4. Total Phenolic Content (TPC)

Total phenolic content of the six species were evaluated using the Folin–Ciocalteu method according to Lee et al. [48]. The samples were prepared in ethanol at a concentration of 1000 μg/mL. Then, 20 μL of the tested samples was added to 100 μL Folin-Ciocalteu’s reagent in 96-well microplates. After 5 min at room temperature, 80 μL of the 7.5% saturated sodium carbonate solution was added and mixed well. The reaction was kept for 30 min and the absorbance was measured at 765 nm. A standard curve of gallic acid was obtained to calculate the total phenolic content and the results were expressed in μg GAE/mg crude extract. All determinations were carried out in six replicates.

### 3.5. 2,2-Diphenyl-1-Picrylhydrazyl (DPPH) Radical Scavenging Assay

Radical scavenging activity using 2,2-diphenyl-1-picrylhydrazyl radical (DPPH) was conducted according to the protocol described by Wan et al. [49]. Samples were prepared at a concentration of 5000 μg/mL, the performing solutions of the extracts were prepared with six serial dilutions made in ethanol (156.25, 312.5, 625, 1250, 2500, and 5000 μg/mL) and quercetin was used as the standard. In a 96-well plate, 50 µL of the test samples was mixed with 100 µL of DPPH (5.9 mg/100 mL MeOH). This was followed by an incubation of the plate in the dark for 30 min. The results were obtained by absorbance measurement at 517 nm and were calculated using the following formula: SC% = [(Ao − As)/Ao] × 100, where Ao is the absorbance of the reagent blank and As is the absorbance of the tested sample. The results were expressed as IC_50_ values in µg/mL, which denote the concentration of sample needed to scavenge 50% of the DPPH free radicals. The IC_50_ for the active samples was calculated from the graph of different concentrations of the extract against percentage inhibition. The IC_50_ values were calculated using the fitted line: Y = a × X + b, IC_50_ = (0.5 – b)/a [18].

### 3.6. α-Glucosidase Inhibitory Activity

The α-glucosidase inhibitory activity was conducted using the procedure reported previously by Lee et al. with minor modifications [48]. Briefly, stock solutions of the crude extracts were prepared in ethanol at a concentration of 5000 μg/mL, and six serial dilution were performed (156.25, 312.5, 625, 1250, 2500, and 5000 μg/mL). The α-glucosidase enzyme (0.02 U/well) and PNPG substrate (1 mM) were prepared in 50 mM phosphate buffer (pH = 6.5). Then, 10 μL of each extract solution was incubated in a 96-well micro plate with 130 μL of the phosphate buffer (30 mM) and 10 μL of enzyme for 5 min at room temperature. The reaction was started by the addition of 50 μL of PNPG to each well (sample, blank substrate, negative control, and positive control). After 15 min of incubation at room temperature, the reaction was stopped by adding 50 μL of 2 M glycine (pH 10). Enzyme activity was quantified by measuring the p-nitrophenol liberated from PNPG at wavelength of 405 nm using a spectrophotometer (SPECTRAmax PLUS, Sunnyvale, CA, USA). The inhibition percentage of inhibition (%) was calculated using the formula: % Inhibition = [(ΔAc – ΔAe)/ΔAc]. The ΔAc is the difference in absorbance between the control (with enzyme) and the blank control (without enzyme), where ΔAe is the difference in absorbance between the sample (with enzyme) and the blank sample (without enzyme). The results were expressed as IC_50_ values in μg/mL.

### 3.7. Nitric Oxide Inhibitory Activity

The nitric oxide inhibition assay was performed according to the method reported by Maulidiani et al. [50]. RAW 264.7 murine macrophage cells acquired from the American Type Culture Collection (ATCC, Rockville, MD) were grown in cell culture flasks in Dulbecco’s Modified Eagle’s Medium (DMEM), which was complemented with 10% fetal bovine serum (FBS) and 1% antibiotic solution (Gibco/BRL) under 5% CO_2_ at 37 °C. When the cells were at 80–90% confluence, cell detachment followed by centrifugation for 10 min in 1000 rpm at 4 °C were performed. A viable cell counting was performed on the Hemocytometer using the Trypan blue cell exclusion of live cells staining technique. A total of 50 μL of cells (1 × 106 cells/mL) was then seeded into a 96-well culture plate with 50 μL of DMEM and incubated for 24 h. The cells were then stimulated by 10 µg/mL of LPS (Escherichia coli, serotype 0111: B4) and 1 µg/mL of IFN-γ in the well with the presence or absence of the extract for 17 h. The sterility of the extract was achieved by filtering the extracts using a 0.2 um filter prior to the assay. The Griess reagent was prepared by adding 1% sulfanilamide and 0.1% N-1-naphthylethylenediamine dihydrochloride in 2.5% H_3_PO_4_, and used to measure the nitrite (NO-2) concentration, according to the stable nitric oxide conversion product. A 50 μL of Griess reagent was mixed with an equal volume of the supernatant of the cultured cells for 15 min at room temperature and in the dark. The absorbance was measured at 550 nm using a Spectramax Plus (Molecular Devices) UV/V microplate reader, the percentage of inhibition was calculated according to Abas et al. [51]. The assay was conducted in six replicates for each extract. The cytotoxicity assay was performed after removing the culture medium and 100 μL of complete DMEM was added to the wells. Subsequently, 20 μL of MTT solution (5 mg/mL in phosphate buffer solution) was added to each well and the plate was incubated for 4 h at 37 °C in a 5% CO_2_ incubator. The medium was then aspirated, and the formed formazan crystals were solubilized by adding 100 μL of DMSO per well and kept for 30 min in the incubator. The intensity of the dissolved formazan crystals (purple color) was measured at 570 nm [29]. The result was expressed as a percentage of cell viability.

### 3.8. Nuclear Magnetic Resonance Measurement

The sample preparation for the NMR measurements was performed according to Kim et al. [52] by adding 0.7 mL of dimethyl sulfoxide-d6 (DMSO) containing 0.1% TSP to 10 mg of crude extracts. The samples were vortexed for 1 min and ultrasonicated for 10 min without heating, followed by centrifugation for 10 min at 10,000 rpm. Approximately 0.6 mL of the supernatant was transferred to an NMR tube for ^1^H-NMR analysis. A 500 MHz Varian INOVA NMR spectrometer (Varian Inc., California, USA) was used at 26 °C. The NMR analysis was performed for all the crude extracts with their six replicates. Each ^1^H-NMR spectrum was collected with a preset acquisition time of 3.53 min, comprising of 64 scans with a spectral width of 20 ppm. Two-dimensional J-resolved (2D JRES) was used as an additional support for the assignment and a confirmation of some compounds. The J-resolved spectrum was acquired in 50 min 18 s using eight scans per 128 increments for the axis of the spi-spin coupling constant, and spectral widths of 66 Hz and 8 K for the chemical shift axis with spectral widths of 5000 Hz.

### 3.9. Multivariate Data Analysis

Chenomx software (v. 5.1, Alberta, Canada) was used to correct the phasing and baseline of all the NMR spectra. The spectral region (δ 0.5–10.0) was binned with a width of δ 0.04, resulting in 237 integral regions per NMR spectrum. The residual signal of DMSO at δ 2.50 was removed from the spectra. The multivariate data analysis (MVDA) was performed using SIMCA-P software (Version 13.0, Umetrics, Umeå, Sweden). Principal component (PCA) and partial least-squares analyses (PLS) were conducted to evaluate the correlation between the activities tested and the metabolites identified. The Pareto scaling was applied in all analysis.

### 3.10. Statistics Analysis

The statistical analysis of TPC, DPPH radical scavenging, α-glucosidase, and nitric oxide inhibitory activities were determined using MS Excel (Version 2010), Minitab software (Version 16, Minitab Inc., State College, PA, USA), and the InStat V2.02 statistical package (GraphPad Software, San Diego, CA, USA). All experiments were performed in six replicates and the results are expressed as mean ± SD. Analysis of variance (ANOVA) was performed to identify significant differences with a probability of *p* < 0.05.

## 4. Conclusions

In this study, the ethanolic extracts at different ratios (0%, 50%, 80%, and 100%) were prepared from the aerial parts of *P. harmala L., Z. album, A. valentinus L., A. leucotrichus, M. vulgare*, and the seeds of *L. albus* were assessed for their α-glucosidase, free radical scavenging (DPPH), and nitric oxide (NO) inhibitory activities as well as the total phenolic contents (TPC). Among the species used in this study, *L. albus* exhibited the highest α-glucosidase inhibitory activity. On the basis of these findings, it can be concluded that the extract obtained from *L. albus* using 100% EtOH was preferable to yield metabolites that might contribute to its α-glucosidase inhibitory activity. ^1^H-NMR metabolomics successfully identified metabolites that were responsible for the activity. In total, 34 metabolites were identified in the ethanolic extract of *L. albus* comprising flavonoids (kaempferol, epicatechin), amino acids (asparagine, thiamine, and proline), vitamin (alpha-tocopherol), fatty acid (linoleic acid), triterpenoids (oleanolic acid, lupeol, and betulinic acid), and isoflavonoids (lupinoisolone A, lupinoisolone C, lupinisol A, and lupinoisoflavone G). The metabolites identified could be used as chemical markers that are responsible for its strong α-glucosidase inhibitory activity exhibited by the ethanolic extract of *L. albus*.

## Figures and Tables

**Table 1:** 2,2-Diphenyl-1-picrylhydrazyl (DPPH) free radical scavenging, α-glucosidase and nitric oxide inhibitory activities of the extracts.

Species	Ethanol Ratio	DPPH Activity		α-Glucosidase Inhibition Activity		Nitric Oxide Inhibition Activity	
		% Inhibition (5000 μg/mL)	IC_50_ μg/mL	% Inhibition (5000 μg/mL)	IC_50_	% Inhibition μg/mL	Cell Viability (5000 μg/mL)
*Peganum harmala* L.	100	10.89 ± 2.62 ^D^_e_	ND	ND	ND	6.44 ± 0.76 ^D^_e_	83.63 ± 0.48
	80	15.42 ± 1.82 ^C^_f_	ND	ND	ND	8.65 ± 0.93 ^C^_e_	90.65 ± 0.65
	50	28.13 ± 1.56 ^B^_c_	ND	ND	ND	11.82 ± 1.22 ^B^_d_	97.43 ± 0.78
	0	44.72 ± 2.56 ^A^_b_	ND	ND	ND	39.73 ± 0.94 ^A^_a_	91.34 ± 0.36
*Anacyclus valentinus*	100	45.99 ± 1.02 ^B^_b_	ND	8. 13 ± 1.07 ^BC^_d_	ND	28.87 ± 1.33 ^C^_c_	67.29 ± 0.38
	80	65.57 ± 1.32 ^A^_b_	25.17 ± 1.62	47.01 ± 2.88 ^A^_b_	ND	40.29 ± 1.14 ^A^_b_	93.11 ± 0.40
	50	31.89 ± 1.12 ^C^_b_	ND	10.13 ± 1.26 ^B^_b_	ND	32.12 ± 1.57 ^B^_b_	97.35 ± 0.61
	0	25.79 ± 1.24 ^D^_c_	ND	7.67 ± 0.07 ^C^_d_	ND	22.06 ± 1.93 ^D^_b_	94.28 ± 0.44
*Zygophyllum album*	100	21.44 ± 1.27 ^B^_d_	ND	ND	ND	27.95 ± 1.34 ^A^_c_	38.79 ± 0.55
	80	19.26 ± 0.88 ^C^_e_	ND	ND	ND	18.37 ± 1.55 ^AB^_d_	96.48 ± 0.63
	50	15.89 ± 0.72 ^D^_d_	ND	ND	ND	13.77 ± 2.04 ^B^_d_	95.81 ± 0.75
	0	48.10 ± 0.66 ^A^_a_	ND	ND	ND	38.28 ± 0.84 ^AB^_b_	67.76 ± 0.71
*Ammodaucus leucotrichus*	100	81.60 ± 0.56 ^A^_a_	26.26 ± 1.22	16.66 ± 1.62 ^B^_c_	ND	50.53 ± 0.62 ^A^_a_	34.52 ± 0.68
	80	50.39 ± 1.76 ^C^_c_	ND	26.60 ± 1.87 ^A^_c_	ND	41.12 ± 1.78 ^C^_b_	99.09 ± 0.22
	50	43.73 ± 2.10 ^B^_a_	ND	4.85 ± 1.15 ^C^_c_	ND	44.94 ± 1.58 ^B^_a_	90.24 ± 0.56
	0	49.80 ± 1.84 ^D^_a_	ND	16.27 ± 0.86 ^B^_c_	ND	39.67 ± 1.88 ^C^_a_	78.55 ± 0.46
*Marrubium vulgare*	100	75.84 ± 2.95 ^A^_a_	19.67 ± 1.33	31.93 ± 2.39 ^C^_b_	ND	48.55 ± 1.46 ^A^_b_	41.74 ± 0.38
	80	78.57 ± 1.88 ^A^_a_	24.08 ± 2.62	68.06 ± 2.15 ^A^_a_	12.66 ± 2.30	49.87 ± 1.32 ^A^_a_	99.63 ± 0.40
	50	35.55 ± 2.38 ^B^_b_	ND	43.43 ± 0.10 ^B^_a_	ND	21.76 ± 0.93 ^B^_c_	98.54 ± 0.39
	0	14.21 ± 1.78 ^C^_d_	ND	20.00 ± 3.00 ^D^_b_	ND	7.77 ± 0.84 ^C^_c_	99.56 ± 0.29
*Lupinus albus*	100	39.59 ± 2.49 ^A^_c_	ND	96.78 ± 1.75 ^A^_a_	6.45 ± 1.30	22.56 ± 0.92 ^AB^_d_	41.74 ± 0.38
	80	36.23 ± 1.67 ^B^_d_	ND	64.57 ± 1.96 ^B^_a_	8.66 ± 1.88	20.44 ± 1.06 ^BC^_c_	99.63 ± 0.40
	50	39.87 ± 1.55 ^C^_c_	ND	45.59 ± 2.76 ^C^_a_	ND	23.04 ± 1.23 ^A^_c_	98.54 ± 0.39
	0	25.70 ± 1.89 ^C^_c_	ND	43.59 ± 2.44 ^C^_a_	ND	19.67 ± 1.56 ^C^_b_	92.43 ± 0.77
*Curcumin* (IC50)	-	-	-	-	-	10.97 ± 0.81	-
*Quercetin*	-	74.84 ± 0.67	12.27 ±1.20	60.80 ± 1.21	8.47 ± 1.57	-	-

Values are the means ± standard deviation based on six replicates. The subscripts represent the comparison between different ethanol ratios in the same species. The superscripts represent the comparison between the same ethanol ratios in the different species. Means with different subscripts and superscripts letters are significantly different (*p* < 0.05). The mean ± standard deviation for DPPH inhibition of standard, quercetin at 50 μg/mL was 74.84 ± 0.67% (IC_50_ = 12.27 ± 1.20 μg/mL). The mean ± standard deviation for α-glucosidase inhibition of standard, quercetin at 50 μg/mL was 60.80 ± 1.21% (IC_50_ = 8.47 ± 1.57 μg/mL). The mean ± standard deviation for NO inhibition of standard, curcumin was (IC_50_ = 10.97 ± 0.81 μg/mL). GAE: gallic acid equivalent; dw: dry weight. ND: not determined.

**Table 2:** Assignments of compounds obtained from the 100% ethanol extract of *L. albus* (s: singlet, d: doublet, t: triplet, dd: doublet of doublet, br s: broad singlet, br t: broad triplet).

Compounds	Chemical Shift	References
(1) Formic acid	8.45 (s)	Erbaş et al., 2005
(2) Adenosine	8.37 (s), 8.23 (s)	-
(3) Hydroxybutyrate	3.58 (m), 2.26 (m), 1.78 (m)	-
(4) Asparagine	2.96 (m), 2.88 (m), 4.02 (dd, 7.5, 4)	-
(5) Proline	4.11 (m), 3.35 (m), 3.30 (m), 2.06 (m), 1.98 (m)	-
(6) Thiamine	9.47 (s), 8.03 (s), 5.47 (s), 3.87 (t, 5.8), 3.20 (t, 5.5), 2.55 (d, 15.5)	-
(7) Epicatechin	7.05 (br s), 6.96 (m), 6.12 (d, 1.5), 6.08 (d,1.5)	-
(8) Caprate	2.15 (br s), 1.46 (br s), 1.25 (br s), 0.86 (br s)	-
(9) Kaempferol	8.02 (d, 8.0), 6.92 (d, 8.0), 6.35 (d, 2.0), 6.20 (d, 2.0)	-
(10) Methoxy flavone	7.16 (d, 2.0), 6.90 (d, 8.5)	-
(11) α-Tocopherol	1.24 (s), 2.05 (m), 1.80 (m), 1.65 (m), 1.52 (m), 1.40 (m), 1.17 (m), 0.88 (d, 6.5)	-
(12) Stearic acid	0.93 (t, 6.5), 1.79 (tt, 7.0), 2.43 (t, 7.5)	-
(13) Palmitic acid	2.37 (t, 4.0), 1.70 (m), 1.47 (m), 0.89 (t, 18.0)	-
(14) Oleic acid	5.35 (m), 2.40 (t, 4.0), 2.03 (m), 1.60 (m), 0.89 (t, 20)	-
(15) Linoleic acid	5.44 (m), 2.91 (t, 6.0), 2.28 (t, 7.0), 2.02 (m), 1.70 (m), 1. 45 (m), 0.93 (t, 6.5)	-
(16) Oleanolic acid	5.14 (br s), 2.89 (m), 2.73 (m), 1.95 (m), 1.84 (d, 6.0), 1.47 (m), 1.32 (m), 1.09 (m), 0.86 (t,12.5)	-
(17) Betulinic acid	4.94 (s), 3.55 (m), 2.75 (m), 2.27 (m), 1.78 (m)	-
(18) Lupeol	1.64 (s), 4.55 (s), 4.66 (s), 2.37 (m), 3.2 (m), 2.22 (d, 5.5)	-
(19) Gallic acid	7.00 (s)	-
(20) Hydroxyiso lupalbigenin	1.65 (s), 1.78 (s), 1.86 (s), 6.68 (s), 8.29 (s), 12.50 (s), 6.56 (d, 8.30), 5.32 (br t,7.0)	-
(21) Wighteon	1.65 (s), 1.78 (s), 6.49 (s), 5.28 (br t), 3.37 (br d)	-
(22) Luteone	1.78 (s), 1.65 (s), 5.32 (br t, 7.0), 3.44 (d, 7.0)	-
(23) Lupisoflavone	3.88 (m), 7.27 (d, 2.0), 6.88 (d, 8.2)	-
(24) Lupinisoflavone A	6.63 (s), 13.30 (s), 8.35 (s), 7.24 (d, 9.0)	-
(25) Lupinoisolone A	1.25 (br s), 1.36 (s), 1.70 (br s), 6.33 (s), 8.18 (s), 13.40 (s), 3.89 (m), 6.88 (d), (5.33 br t, 7.0), 3.38 (br d, 6.8)	-
(26) Lupinoisolone C	1.28 (s), 1.62 (s), 1.77 (br s), 7. 94 (s), 13.41 (s), 6.41 (d,8.0), 6.44 (br s), 7.03 (d, 8.2), 5.31 (br t, 7.1), 3.75 (br t, 7.0), 3.35 (br d, 7.0)	
(27) Lupinisol A	1.72 (s), 1.89 (s), 6.45 (s), 7.33 (s), 8.12 (s), 13.58 (s), 4.72 (brs), 4.95 (br s), 3.00 (m), 4.4 (m), 7.29 (dd, 8.3, 2.0), 6.89 (d, 8.3), 5.35 (br t,7.0), 3.35 (br d, 7.0)	Tahara et al., 1984
(28) Lupinisol B	6.52 (s), 8.18 (s), 13.00 (s), 4.77 (br s), 4.94 (br s), 6.95 (d, 8.0), 5.32 (br t, 7.0), 4.48 (dd, 7.0,4.0), 3.48(br d,5.0)	
(29) Lipinisol C	8.14 (s), 6.56 s, 13.01 (s), 4.85 (br s), 5.04 (br s), 7.05 (d, 7.0), 5.28 (br t, 7.3), 4.37 (br d, 5.0), 3.38 (br d, 5.0)	-
(30) Chandalone	1.47 (s), 6.32 (s), 8.14 (s), 13.46 (s),1.25 (br s), 7.35 (d, 2.0), 7.29 (dd, 2.0, 7.0) 6.88 (d, 2.0), 6.69 (d, 9.0), 5.79 (d, 10.0), 5.33 (br t), 3.38 (br, 5.5)	
(31) Isoderrone	1.41 (s), 8.25 (s), 13.00 (s), 7.29 (d, 2.0), 6.48 (d, 2.0), 6.44 (d, 1.5) 6.31 (d, 1.5), 5.77 (d, 5.0)	-
(32) Lupinalbin F	1.72 (s), 1.89 (s), 7.33 (s), 8.12 (s), 13.58 (s), 4.72 (br s), 4.95 (br s), 3.00 (m), 4.40 (m), 7.29 (dd, 8.3, 2.0), 6.89 (d, 8.3), 6.45 (s), 5.35 (br t, 7.0), 3.35 (br d, 7.0)	
(33) Lupinoisoflavone G	1.25 (s), 1.29 (s), 6.37 (s), 8.14 (s), 1.73 (br s), 7.35 (d, 2.0), 7.29 (dd, 8.0,2.0). 5.33 (br t, 7.1), 3.18 (br d, 8.0)	-
(34) Genistein	6.39 (s), 7.69 (s), 7.32 (d, 7.0), 6.33 (s), 6.95 (d, 7.5)	
(35) Unknown	1.24 (s)	-
(36) Unknown	5.22 (s)	
